# Identifying Pregnant Women With Disabilities and Maternal and Newborn Outcomes

**DOI:** 10.1001/jamanetworkopen.2025.2159

**Published:** 2025-03-27

**Authors:** Alka Dev, Willi Horner-Johnson, Andrew Schaefer, Cecilia Ganduglia-Cazaban, Thérèse A. Stukel, David C. Goodman, JoAnna K. Leyenaar

**Affiliations:** 1 The Dartmouth Institute for Health Policy and Clinical Practice, Geisel School of Medicine at Dartmouth, Hanover, New Hampshire; 2 Oregon Health and Science University Institute on Development and Disability, Portland; 3 Center for Healthcare Data, UTHealth Houston School of Public Health, Houston, Texas; 4 ICES, Toronto, Ontario, Canada; 5 Institute of Health Policy, Management and Evaluation, University of Toronto, Toronto, Ontario, Canada; 6 Department of Pediatrics, Geisel School of Medicine at Dartmouth, Hanover, New Hampshire

## Abstract

**Importance:**

Pregnant women with disabilities are at higher risk of poor pregnancy and birth outcomes. Different methods for identifying disability may affect estimates of health disparities in this population.

**Objective:**

To compare pregnancy and birth outcomes among pregnant women using different ways of identifying maternal disability.

**Design, Setting, and Participants:**

Retrospective cohort study of linked vital records and maternal and newborn claims for Medicaid-insured live births from January 2010 to December 2014 in Texas. Data analysis was conducted from October 2023 to May 2024.

**Exposure:**

Births grouped into 5 maternal cohorts: no identified disability, disability benefits enrollment only, disability diagnostic code only, both benefits enrollment and a diagnostic code, and either disability benefits or a diagnostic code.

**Main Outcomes and Measures:**

Mode of delivery (cesarean) and severe maternal morbidity (SMM) were identified from maternal claims. Low birthweight (LBW), preterm birth (PTB), and small for gestational age (SGA) were identified from birth certificates. Modified Poisson regression with robust variance estimators was used to estimate adjusted risk ratios (aRRs) for the association of each of the 5 outcomes with disability group status.

**Results:**

Among 921 218 births (mean [SD] maternal age at birth, 25.1 [5.7] years), 895 201 (97.2%) were to mothers with no disability, 6160 (0.7%) were to mothers enrolled in disability benefits only, 17 742 (1.9%) were to mothers with a disability diagnostic code only, 2115 (0.2%) were to mothers with both benefits enrollment and a disability code, and 26 017 (2.8%) were to mothers meeting either disability definition. Compared with those without disabilities, those with only disability diagnostic codes had the highest rates for cesarean delivery (306 589 births [34.3%] vs 7658 births [43.2%]), LBW (750 058 births [8.4%] vs 869 births [14.2%]), and PTB (92 807 births [10.4%] vs 977 births [15.9%]). Compared with those with no disability, the adjusted relative risks were highest in the diagnostic codes only group for cesarean delivery (aRR, 1.22; 95% CI, 1.20-1.24), LBW (aRR, 1.77, 95% CI, 1.71-1.84), and PTB (aRR, 1.68; 95% CI, 1.62-1.74). The risk for SMM (aRR, 4.82; 95% CI, 3.96-5.86) and SGA (aRR, 1.43; 95% CI, 1.24-1.66) were highest in those with both benefits enrollment and a disability code.

**Conclusions and relevance:**

In this cohort study, disability was associated with adverse outcomes, regardless of definition. However, the burden of disparities was dependent on how disability was defined, suggesting that the assessment of disability-associated health risks should consider how disability is conceptualized.

## Key Points

**Question:**

Are there differences in the characteristics and outcomes of live births to pregnant women with disability receiving disability benefits vs those identified through disability-associated diagnostic codes?

**Findings:**

In this cohort study of 921 218 births, there was little overlap between the disability groups identified using diagnostic codes and through benefits enrollment. Risk of preterm birth and low birthweight were higher in the group based on diagnostic codes, and the risk of severe maternal morbidity and being small for gestational age were higher in those with both diagnostic codes and benefit enrollment.

**Meaning:**

These findings suggest that the burden of adverse pregnancy and birth outcomes among women with disabilities varies by disability definition.

## Teaser

This cohort study analyzes pregnancy and birth outcomes among pregnant women with disabilities identified using diagnostic codes and/or benefits enrollment.

## Introduction

Approximately 1 in 5 women who have given birth in the US report having at least 1 disability.^1^ Pregnancy can be a particularly vulnerable time among those with disabilities, with higher risks of adverse pregnancy and birth-related outcomes, such as cesarean delivery, severe maternal morbidity (SMM), preterm birth (PTB), low birthweight (LBW), and small for gestational age (SGA).^1,2,3,4,5,6^ People with disabilities have been recently recognized by the National Institutes of Health as a population at particular risk of health disparities.^7^ Accurate methods for measuring disability during pregnancy are important to capture the scale of maternal and newborn health disparities borne by this population and guide clinical and policy responses.

Disability can be measured using participant disclosure in survey data, clinician use of disability-related diagnostic codes in hospital discharge data and administrative claims, or eligibility for disability-related entitlements based on federal- and state-level evaluations such as the supplemental security income or social security disability insurance programs.^8,9,10^ These approaches yield distinct and partially overlapping cohorts. For instance, in a national survey of US adults, 26.8% of respondents indicated some form of disability, with 11.7% reporting multiple types of disability.^11^ Comparatively, one-third of Medicaid enrollees self-identified as disabled, although only 11% of Medicaid beneficiaries were provided coverage based on supplemental security income and social security disability insurance assessments.^12^

The definition of disability in administrative claims data are typically operationalized through systems such as diagnostic codes, encompassing a range of physical, mental, sensory, and cognitive conditions.^9^ Medicaid enrollment records offer the added advantage of identifying individuals who have qualified for state and federal benefits, including public health insurance, based on disability. Analyzed together, Medicaid enrollment and claims data offer a unique opportunity to compare characteristics and outcomes among Medicaid-insured beneficiaries based on a disability-related diagnosis during pregnancy vs receipt of disability benefits. Therefore, in this study, we assessed how demographic characteristics, comorbid conditions, cesarean delivery, SMM, PTB, LBW, and SGA differed between pregnant women with disabilities identified through diagnostic codes and those qualifying for Medicaid due to disability benefits.

## Methods

### Data Source and Study Cohort

**Figure 1.**
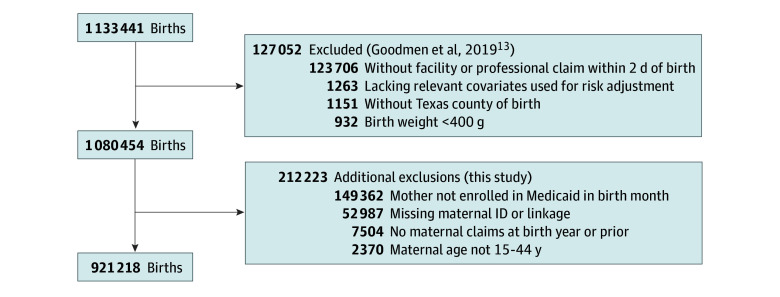
Sample Selection ID indicates identification.

This retrospective cohort study was approved by the institutional review boards of Dartmouth College, the University of Texas Health Science Center at Houston, and the Texas Health and Human Services Commission,^13^ with a waiver of informed consent per 45 CFR 46.116(d). It followed the Strengthening the Reporting of Observational Studies in Epidemiology (STROBE) reporting guidelines for cohort studies. The dataset included all newborns born in Texas and insured by Medicaid during the period January 1, 2010, through December 31, 2014, with linkages to newborn and maternal Medicaid enrollment files, natality and mortality vital records, and maternal and newborn facility (ie, hospital) and professional claims and encounters. Although Texas did not expand Medicaid under the Affordable Care Act, it does provide Medicaid coverage to pregnant women with incomes up to 198% of the federal poverty level, placing it at the halfway point compared with other states.^14,15^ The Goodman team^13^ initially applied exclusions (Figure 1). To this, we applied additional exclusions, including births missing maternal-infant linkage (52 987 births), mothers not enrolled in Medicaid in the birth month (149 362 births), mothers missing claims at birth or in the year prior (7504 births), and mothers not between ages 15 to 44 years (2370 births). Thus, our analytical cohort consisted of births to mothers aged 15 to 44 years who were enrolled in Medicaid during the birth month, had a birth hospitalization record, and had 2 or more health care claims during the preceding 12 months.

### Disability Group Definitions

STAR+ is a Texas Medicaid managed care program that provides health insurance and long-term services and support, including pregnancy, labor, delivery, and postpartum coverage for Texan adults with disabilities.^16^ To qualify for Medicaid disability benefits, individuals must pass strict disability, financial, and employment assessments from the supplemental security income program. This process is stringent and burdensome, potentially excluding many disabled people.^17^ People in this group qualify for Medicaid due to disability. For the diagnostic code–based disability definition, we used disability-associated *International Classification of Diseases, Ninth Revision *(*ICD-9*) codes previously published for the determination of maternal disability in administrative claims.^2^ These codes were drawn from published algorithms and then vetted by a group of 13 clinicians with disability expertise to select codes judged to be indicative of physical, sensory, intellectual, or developmental disabilities.^2^ Women in this group likely qualify for Medicaid due to pregnancy.

We created 4 mutually exclusive groups of mothers as defined in the Box: (1) no identified disability; (2) STAR+ only, which included those eligible for disability benefits and Medicaid but who did not have any *ICD-9* disability codes^14^; (3) *ICD-9* only, which included those with *ICD-9* disability codes at 2 or more prenatal care visits or during at least 1 emergency department visit or hospitalization but who were not enrolled in disability benefits; and (4) both, which included births to mothers with both disability-based eligibility and relevant *ICD-9* disability codes. We also combined groups 2 to 4 to create a fifth overlapping group of mothers meeting either definition.

Box. **Disability Group Definitions**

1. No disability: Mother not eligible for Medicaid due to a disability with no *International Classification of Diseases, Ninth Revision (ICD-9)* codes pertaining to disability
2. STAR+ only: Mother enrolled in the Texas STAR+ Medicaid program
3. *ICD-9* only: Mother had at least 1 disability associated *ICD-9* code over 2 prenatal care visits, or 1 emergency department visit or 1 hospitalization
4. Both STAR+ and *ICD-9*: Mother was in the STAR+ program and met the *ICD-9* diagnostic criteria
5. Either: Mother met any disability definition (combined definitions 2, 3, and 4)

### Sociodemographic Characteristics

Maternal sociodemographic characteristics, self-reported on the birth certificate, included age, education, race, ethnicity, and marital status. Education categories included less than high school, high school, and some college or higher. Race categories included American Indian or Alaska Native, Asian, Black, Pacific Islander, White, other (defined as any race not otherwise specified), more than 1 race, and unknown. Due to small numbers, we combined American Indian and Alaska Native, Pacific Islander, other, and unknown race categories for our analyses. Hispanic ethnicity was measured separately; thus, mothers could identify as Hispanic and designate a race. Race and ethnicity were included to account for disparities. Marital status referred to being married or not at the time of birth. We also examined smoking during pregnancy, given the disparity in smoking rates among pregnant women with and without disabilities.^1,18^

### Clinical Characteristics

We determined multiple gestations (twins or more) from claims data, parity (number of prior live births) from birth certificates, and receipt of prenatal care (yes or no) from birth certificates. The Elixhauser Comorbidity Index was applied to identify chronic conditions that may complicate pregnancies among those with STAR+ eligibility who were otherwise missing *ICD-9* codes associated with disability. We excluded codes that overlapped with the *ICD-9* disability algorithm. The index is based on 30 *ICD-9* diagnostic categories for comorbid conditions such as diabetes, hypertension, heart failure, and other chronic diseases.^19^ It has been validated in various populations to predict patient outcomes and health care utilization.^20^ We also created a binary maternal mental health variable using previously published *ICD-9* codes to identify severe mental illness associated with disability such as schizophrenia, bipolar disorder, depression, and anxiety.^5^ The Elixhauser index and the maternal mental health variable were used for descriptive analyses only.

### Maternal and Newborn Outcomes

Five maternal and newborn outcomes were assessed: (1) cesarean delivery, as reported on the birth certificate; (2) SMM (defined per Centers for Disease Control and Prevention guidelines), including any of 21 conditions and blood transfusions occurring during the birth hospitalization,^21^ as reported in maternal professional and facility claims; (3) LBW, defined as a newborn weighing less than 2500 g at birth,^22^ as reported on the birth certificate; (4) PTB, defined as a birth occurring at less than 37 weeks’ gestation,^23^ as reported on the birth certificate; and (5) SGA, defined as a birth weight of less than the 10th percentile for gestational age and sex using the International Fetal and Newborn Growth Consortium for the 21st Century standards and derived from birth certificate data.^24^

### Statistical Analysis

Proportions and frequencies were used to characterize sociodemographic characteristics for the overall sample and by disability group. We used modified Poisson regression with robust variance estimators to estimate adjusted risk ratios (aRRs) for the association of each of the 5 outcomes with disability group status, with clustering at the level of mothers for multiple newborns. There were 2 sets of regression models—one set comparing the mutually exclusive groups (2-4) with those with no identified disability (group 1), and the second set comparing outcomes in those who met any disability definition (group 5) with those with no identified disability. The unit of analysis was the individual live birth. Potential confounding variables included in regression models included maternal age, race, ethnicity, educational attainment, marital status, parity, multiple gestations, and smoking during pregnancy. All *P* values were 2-sided, with a value less than or equal to .05 considered statistically significant. Data analysis was conducted with SAS version 9.4 (SAS Institute) from October 2023 to May 2024.

## Results

### Sociodemographic Characteristics

**Figure 2.**
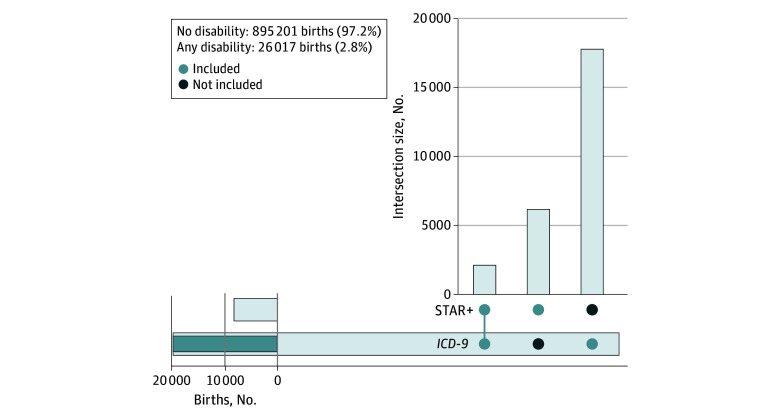
Upset Plot of the Intersection of *International Classification of Diseases, Ninth Revision (ICD-9) *and STAR+ Groups The horizontal bar chart reflects the total number of births of STAR+ and *ICD-9* groups. The vertical bar chart reflects 3 intersections of data: (1) the STAR+ and *ICD-9* group, the STAR+ only group, and the *ICD-9* only group.

Our study consisted of 921 218 births, of whom 19 037 (2.1%) were to Asian mothers, 139 509 (15.1%) were to Black mothers, and 623 009 (67.6%) were to White mothers (Table 1). Additionally, 534 900 mothers (58.1%) identified as being Hispanic. Among all births, the mean (SD) maternal age at birth was 25.1 (5.7) years and 616 922 births (67.0%) were to mothers with a high school education or less. The benefits enrollment group (STAR+) had the highest proportion of Black mothers (2216 births [36.0%]) and those with a high school education or less (5055 births [82.2%]) and the lowest proportion of married mothers (1481 births [24.0%]). The no disability group had the largest proportion of Hispanic mothers (522 883 births [58.4%]). Disaggregating maternal disability, 895 201 births (97.2%) were to mothers with no identified disability (group 1), 6160 births (0.7%) were to mothers enrolled in STAR+ with no *ICD-9* codes for disability (group 2), 17 742 (1.9%) births were to mothers with only *ICD-9* codes for disability (group 3), 2115 births (0.2%) were to mothers with both STAR+ enrollment and *ICD-9* disability codes (group 4), and 26 017 (2.8%) births were to women meeting either disability definition (group 5). See Figure 2 for the intersection of STAR+ and *ICD-9* groups.

**Table 1.** Texas Medicaid Livebirths and Maternal Disability Cohort, 2010 to 2014

Characteristic	Births by disability cohort, No. (%)					
	No disability (n = 895 201)	Only STAR+ (n = 6160)	Only *ICD-9 *(n =17 742)	Both STAR+ and *ICD-9 *(n = 2115)	(Either STAR+ or *ICD-9 *(n =26 017)^*a*^	Total (N = 921 218)
Mothers, No.	749 634	5149	14 797	1868	21 824	771 448
Maternal characteristics						
Age, mean (SD), y	25.1 (5.7)	25.2 (5.5)	25.3 (5.9)	25.9 (5.9)	25.3 (5.8)	25.1 (5.7)
Education						
<High school	271 057 (30.3)	2656 (43.1)	4919 (27.7)	801 (37.9)	8376 (32.2)	279 433 (30.3)
Completed high school	327 503 (36.6)	2410 (39.1)	6760 (38.1)	816 (38.6)	9986 (38.4)	337 489 (36.6)
>Some college	296 641 (33.1)	1094 (17.8)	6063 (34.2)	498 (23.6)	7655 (29.4)	304 296 (33.0)
Race						
Asian	18 757 (2.1)	47 (0.8)	205 (1.2)	28 (1.3)	280 (1.1)	19 037 (2.1)
Black	133 800 (15.0)	2216 (36.0)	2927 (16.5)	566 (26.8)	5709 (21.9)	139 509 (15.1)
White	606 222 (67.7)	3126 (50.6)	12 422 (70.0)	1239 (58.6)	16 787 (64.5)	623 009 (67.6)
>1 race	11 520 (1.3)	111 (1.8)	287 (1.6)	30 (1.4)	428 (1.6)	11 948 (1.3)
Other^b^	124 902 (14.0)	660 (10.7)	1901 (10.7)	252 (11.9)	2813 (10.8%)	127 715 (13.9)
Hispanic ethnicity	522 883 (58.4)	2673 (43.4)	8.292 (46.7)	1052 (49.7)	12 017 (46.2)	534 900 (58.1)
Married	339 221 (37.9)	1481 (24.0)	6177 (34.8)	655 (31.0)	8313 (32)	347 534 (37.7)
Parity						
1	337 038 (37.7)	2010 (32.7)	6902 (38.9)	818 (38.7)	9730 (37.4)	346 768 (37.7)
2	253 483 (28.3)	1612 (26.2)	4803 (27.1)	527 (24.9)	6942 (26.7)	260 425 (28.3)
≥3	304 488 (34.0)	2534 (41.2)	6034 (34.0)	770 (36.4)	9338 (35.9)	313 826 (34.1)
Receipt of prenatal care	857 621 (96.8)	5765 (95.3)	16 954 (96.6)	1981 (94.8)	24 700 (94.9)	882 321 (96.8)
No. of visits, mean (SD)	10.3 (3.6)	10.1 (4.0)	10.6 (4.1)	10.2 (3.9)	10.5 (4.1)	10.3 (3.6)
Mental health disorder	54 357 (6.07)	2846 (46.2)	4302 (24.2)	1094 (51.7)	8242 (31.7)	62 599 (6.80)
Smoking during pregnancy	60 900 (6.8)	671 (10.9)	1976 (11.1)	169 (8.0)	2816 (10.8)	63 716 (6.9)
Cesarean delivery	306 589 (34.3)	2545 (41.3)	7658 (43.2)	877 (41.5)	11 080 (42.6)	317 669 (34.5)
SMM with transfusion	19 712 (2.2)	291 (4.7)	1057 (6.0)	188 (8.9)	1536 (5.9)	21 248 (2.3)
Any SMM without transfusion	8324 (0.9)	175 (2.8)	741 (4.2)	142 (6.7)	1058 (4.1)	9382 (1.0)
Newborn characteristics						
Sex						
Female	437 336 (48.9)	2985 (48.5)	8629 (48.6)	1034 (48.9)	12 648 (48.6)	449 984 (48.9)
Male	457 865 (51.1)	3175 (51.5)	9113 (51.4)	1081 (51.1)	13 369 (51.4)	471,234 (51.1)
Multiple gestation	19 152 (2.1)	176 (2.9)	523 (3.0)	42 (2.0)	741 (2.8)	19 893 (2.2)
Preterm (<37 wk)	92 807 (10.4)	977 (15.9)	3253 (18.3)	372 (17.6)	4602 (17.7)	97 409 (10.6)
Birthweight, g						
500-1499	10 878 (1.2)	133 (2.2)	469 (2.6)	48 (2.3)	650 (2.5)	11 528 (1.3)
1500-2499	64 180 (7.2)	736 (12.0)	2368 (13.4)	264 (12.5)	3368 (12.9)	67 548 (7.3)
≥2500	820 143 (91.6)	5291 (85.9)	14 905 (84.0)	1803 (85.3)	21 999 (84.6)	842 142 (91.4)
Small for gestational age	45 822 (5.1)	488 (7.9)	1323 (7.5)	180 (8.5)	1991 (7.7)	47 813 (5.2)

Abbreviations: *ICD-9, International Classification of Diseases, Ninth Revision*; SMM, severe maternal morbidity.

^a^ This group is not mutually exclusive from the others because it includes those who were either in the only STAR+ group, the only *ICD-9* group, or the both STAR+ and *ICD-9 *group.

^b^ Includes 2223 American Indian or Alaska Native mothers, 1116 Pacific Islander mothers, 2341 mothers with unknown race from birth certificate data, and 122 035 mothers who self-identified as other race (ie, any race not otherwise specified).

### Clinical Characteristics

Compared with those without disabilities, those with only disability diagnostic codes had the highest rates for cesarean delivery (306 589 individuals [34.3%] vs 7658 births [43.2%]), LBW (750 058 births [8.4%] vs 869 births [14.2%]), and PTB (92 807 births [10.4%] vs 977 births [15.9%]). The STAR+ group had the highest proportion of mothers with 3 or more prior live births (2534 births [41.2%]). The majority (95% or more) of mothers in every group received prenatal care, with a mean of more than 10 visits (Table 1). For groups with *ICD-9* codes, the 30 most common disability-associated codes, organized by type of disability, are presented in eTable 1 in Supplement 1. Epilepsy-related codes were the most prevalent (6417 births [36.2%]), followed by rheumatoid arthritis (1054 births [6.0%]) and unspecified hearing loss (950 births [5.4%]). All other conditions were observed in less than 2% of the disability cohorts. Neurological disorders were most prevalent overall.

Obesity, chronic pulmonary disease, diabetes, deficiency anemias, drug use, and depression were more prevalent in all disability groups compared with the no disability group (eTable 2 in Supplement 1). Prevalence of maternal psychoses was much higher for mothers receiving disability benefits; it affected 1490 births (24.2%) in the STAR+ only group and 547 births (25.9%) in the STAR+ with *ICD-9* codes group compared with 1480 births (8.3%) in the *ICD-9* only group, and 12 431 births (1.4%) in the no disability group.

### Adjusted Associations of Disability With Maternal and Newborn Outcomes

The aRRs for outcomes are presented in Table 2. The risk of cesarean delivery was significantly higher for STAR+ only (aRR, 1.12; 95% CI, 1.08-1.16), *ICD-9* only (aRR, 1.22; 95% CI, 1.20-1.24), and those meeting either disability definition (aRR, 1.17; 95% CI, 1.16-1.19) compared with the no disability group. For all other outcomes, the risk was higher across all disability groups when compared with those with no disability. For most outcomes, the magnitude of excess risk was lowest in the STAR+ only group and highest in the *ICD-9* only group. The aRRs of SMM ranged from 2.59 (95% CI, 2.15-3.12) in the STAR+ only group to 4.82 (95% CI, 3.96-5.86) in the group meeting both STAR+ and *ICD-9* diagnostic code definitions. For PTB, the aRRs ranged from 1.32 (95% CI, 1.24-1.39) for the STAR+ only group to 1.68 (95% CI, 1.62-1.74) in the *ICD-9* only group. For LBW, the aRRs ranged from 1.36 (95% CI, 1.27-1.45) for the STAR+ only group to 1.77 (95% CI, 1.71-1.84) in the *ICD-9* only group. The relative risk of SGA was highest among those with both STAR+ enrollment and *ICD-9* codes (aRR, 1.43; 95% CI, 1.24-1.66). The complete models for each outcome are available in eTable 3 in Supplement 1.

**Table 2.** Risk of Maternal and Newborn Outcomes With Fully Adjusted Models

Outcome	Adjusted risk ratio (95% CI)^a^				
	No disability	Only STAR+	Only *ICD-9*	Both STAR+ and *ICD-9*	Either STAR+ or *ICD-9*
Cesarean delivery	1 [Reference]	1.12 (1.08-1.16)	1.22 (1.20-1.24)	0.97 (0.92-1.02)	1.17 (1.16-1.19)
Severe maternal morbidity	1 [Reference]	2.59 (2.15-3.12)	4.58 (4.16-5.04)	4.82 (3.96-5.86)	4.12 (3.79-4.47)
Preterm birth	1 [Reference]	1.32 (1.24-1.39)	1.68 (1.68-1.74)	1.49 (1.36-1.64)	1.57 (1.53-1.62)
Low birthweight	1 [Reference]	1.36 (1.27-1.45)	1.77 (1.71-1.84)	1.51 (1.36-1.67)	1.64 (1.6-1.69)
Small for gestational age	1 [Reference]	1.26 (1.15-1.38)	1.38 (1.31-1.46)	1.43 (1.24-1.66)	1.35 (1.29-1.41)

Abbreviation: *ICD-9, International Classification of Diseases, Ninth Revision.*

^a^ Adjusted for age, race, ethnicity, education, marital status, parity, multiple gestations, and smoking.

## Discussion

To our knowledge, this cohort study is the first to evaluate the characteristics and outcomes of women receiving Medicaid disability benefits compared with those identified using disability-related diagnostic codes. Women receiving disability benefits were more likely to be Black and have less than a high school education, suggesting they were more socially disadvantaged than other Medicaid enrollees. When disability diagnostic codes were present, more than one-third of births involved maternal epilepsy. Women in all disability groups experienced several comorbidities; maternal psychosis was most common among those receiving disability benefits. The risk of SMM, LBW, and PTB were highest among those with only disability-related diagnostic codes. The risk for SGA was highest among those with both disability benefits and disability diagnostic codes. Our results suggest that disability, across all definitions, conferred some disadvantage with respect to these outcomes and was most pronounced among those with diagnostic codes but no disability benefits.

The prevalence of disability identified using diagnostic codes is highly variable depending on the data source, disability codes or questions used, and the population under study. For example, using self-reported disability in surveys alone yields a range of 6.3% to 23.3% prevalence in working-age adults, a process that is highly sensitive to survey methods, even with the same questions.^25^ We found that 2% of births were to mothers who had a diagnostic code for disability. Other studies using hospital discharge data across 13 US states have documented diagnostic code–based disability prevalence to be lower (<1%).^26,27^ The sensitivity of using disability codes from hospital discharge data with all insurance types has been found to be low compared with electronic health records, suggesting that identifying disability at single points of observation could result in an underestimation of disability prevalence.^28^ Our criterion differed in that it included multiple clinical encounters with a disability diagnosis. Using a longer observation period for mothers, investigators in Canada yielded a much higher prevalence of any disability (11.1%) for all Ontario births than our findings.^2^ The estimate from Ontario is closer to what is found in the US from self-reported disability responses in population-based surveys.^25^ We found a slightly lower prevalence among pregnant women enrolled in Medicaid due to a disability (0.9%) than was recorded in disability-eligible pregnant Medicaid recipients in Texas in 1997 (1.1%).^29^ As a proportion of all Medicaid-insured births, the lower disability prevalence in our study could be due to a higher proportion of Hispanic mothers who are less likely to receive disability benefits than their White and Black counterparts.^30^ The prior study^30^ did not report frequencies by race or ethnicity so we could not verify this assumption.

Similar to other studies, we found higher rates of multiple chronic comorbidities among women with disabilities across each disability group, suggesting high health service needs.^31,32^ The risk of an adverse pregnancy or birth outcome was higher for pregnant women who met any of our disability definitions relative to those without an identified disability, as has been reported in comparable studies. Among California births using a similar diagnosis-based disability definition to ours, investigators reported a 4 times higher risk of SMM among those with any diagnosed disability compared with those with no disability,^6^ similar to our finding of 4.6 times the risk of SMM among those with disability-related diagnostic codes. Similarly, while SMM rates were lower in Ontario birth data, disparities in comparison with nondisabled women were present across different types of disability.^2^ For newborn outcomes, our findings of elevated risk for PTB, LBW, and SGA were also comparable to existing evidence.^25,31,32^ For example, using data from 12 clinical sites across 19 hospitals in the US,^27^ 14 investigators found that births to women with any disability had nearly twice the risk of PTB or LBW and a higher risk of SGA compared with those with no disability, similar to our estimate or 1.6 for PTB and LBW, and 1.4 for SGA. The high prevalence of psychosis among those who are enrolled in the STAR+ program suggests that mental illness could be an important eligibility criterion for Medicaid. Similar rates of mental illness have been reported among people with disabilities in the US and Canada.^33,34^

Our findings have 3 important implications. First, our data suggest that those with disability-related diagnostic codes may be at the highest risk for adverse outcomes, regardless of their disability benefits status, and require ongoing efforts to be identified appropriately during prenatal care. The Social Security Administration states that its process has evolved to prioritize the functional assessment of disability benefit applicants as opposed to granting benefits based on the listing of impairments.^35^ However, people with disabilities assert that the administrative burdens of applying and qualifying for disability benefits are too onerous to overcome, leaving many who would qualify with no disability-specific benefits.^17^ Such individuals could be identified in administrative data through diagnostic codes during pregnancy. Second, for those with disabling conditions who are not receiving disability program benefits and qualify for Medicaid due to pregnancy, having multiple comorbidities and obstetric complications would pose a serious health disadvantage upon losing coverage after pregnancy in states that have not expanded Medicaid under the Affordable Care Act. Losing insurance coverage could result in higher morbidity and mortality, adversely affecting both maternal and infant well-being.^36^ Third, including recipients of STAR+ disability benefits allowed us to identify an additional group of pregnant women with disabilities that experienced substantial disadvantages and disparities in outcomes compared with those with no disabilities. This group would be overlooked if we relied on diagnostic codes alone. Utilizing disability benefit enrollment and diagnosis data can yield a more comprehensive disability sample for analyses using Medicaid data. Disability is a multifaceted concept encompassing both medical and social models, and disability identification should be an important consideration for disability policy reform.

### Limitations

This study has certain limitations. First, we did not have access to prepregnancy claims and could not verify prepregnancy disability. We also could not verify Medicaid eligibility criteria beyond STAR+ enrollment. Second, some of the mothers in our sample may have had disability-related diagnoses that were not reflected in Medicaid claims or benefits enrollment, resulting in their misclassification. Additionally, we may be underidentifying births to women with disabilities because we were relying on diagnoses in perinatal data rather than full medical records. Third, we were only able to assess outcomes regarding women with disabilities in administrative data, although neither diagnoses nor qualification for benefits constitutes an ideal mechanism for identifying disability. Fourth, we were limited by the fact that the cohort was initially constructed to assess neonatal outcomes, and our data only included linkages to live births among those insured by Medicaid. Thus, these findings may not be generalizable to mothers and newborns insured by commercial insurance or with different outcomes. For example, we could not measure fetal losses.

## Conclusions

In this cohort study of disability and birth outcomes, all definitions of disability identified women with a higher risk of adverse maternal and neonatal outcomes compared with those with no indication of disability. Disability-related diagnostic codes and receipt of disability benefits identified different samples of individuals, albeit with some overlap. Utilizing both approaches provided the most inclusive option for identifying births to women with disabilities.

## Notes

Supplement 1. **eTable 1.** 30 Most Common Disability-Associated ICD-9 Codes in Our Sample **eTable 2.** Most Prevalent Elixhauser Illnesses **eTable 3.** Complete Regression Models for Each Outcome

Supplement 2.
Data Sharing Statement
