# Supplementary material for: Identifying Pregnant Women With Disabilities and Maternal and Newborn Outcomes
Source: JAMA Netw Open. 2025 Mar 27;8(3):e252159. doi: 10.1001/jamanetworkopen.2025.2159 (PMC11950893; doi:10.1001/jamanetworkopen.2025.2159)
Supplement: Supplement 1. — eTable 1. 30 Most Common Disability-Associated ICD-9 Codes in Our Sample eTable 2. Most Prevalent Elixhauser Illnesses eTable 3. Complete Regression Models for Each Outcome [file jamanetwopen-e252159-s001.pdf]

Supplemental Online Content

Dev A, Horner-Johnson W, Schaefer A, et al. Identifying pregnant people with disabilities and maternal and newborn outcomes. *JAMA Netw. Open*. 2025;8(3):e252159.
doi:10.1001/jamanetworkopen.2025.2159

eTable 1. 30 Most Common Disability-Associated *ICD-9* Codes in Our Sample

eTable2. Most Prevalent Elixhauser Illnesses

eTable 3. Complete Regression Models for Each Outcome

This supplemental material has been provided by the authors to give readers additional information about their work.

© 2025 Dev A et al. *JAMA Network Open*.

eTable 1: 30 Most Common Disability-Associated *ICD-9* Codes in Our Sample

	Only ICD-9 dx		Both STAR+ and ICD9 dx	
	**N**	**%**	**N**	**%**
**PHYSICAL DISABILITY: Neurological Disorders**				
345.90--Epilepsy, unspecified, without mention of intractable epilepsy	4,581	25.82%	526	24.87%
345.10—Generalized convulsive epilepsy, without mention of intractable epilepsy	1,174	6.62%	136	6.43%
345.40--Localization-related (focal) (partial) epilepsy and epileptic syndromes with complex partial seizures, without mention of intractable epilepsy	453	2.55%	68	3.22%
343.9--Infantile cerebral palsy, unspecified	347	1.96%	110	5.20%
345.80--Other forms of epilepsy and recurrent seizures, without mention of intractable epilepsy	340	1.92%	47	2.22%
345.00--Generalized nonconvulsive epilepsy, without mention of intractable epilepsy	259	1.46%	39	1.84%
340--Multiple sclerosis	253	1.43%	39	1.84%
333.94--Restless legs syndrome (RLS)	199	1.12%	27	1.28%
356.9--Unspecified hereditary and idiopathic peripheral neuropathy	289	1.63%	64	3.03%
355.9--Mononeuritis of unspecified site	275	1.55%	73	3.45%
**TOTAL Neurological Disorders**	**8170**	**46.05%**	**1129**	**53.38%**
**PHYSICAL DISABILITY: Musculoskeletal Disorders**				
714.0--Rheumatoid arthritis	883	4.98%	171	8.09%
722.10--Displacement of lumbar intervertebral disc without myelopathy	643	3.62%	145	6.86%
722.52--Degeneration of lumbar or lumbosacral intervertebral disc	428	2.41%	128	6.05%
720.2--Sacroiliitis, not elsewhere classified	402	2.27%	25	1.18%
715.90-- Osteoarthrosis, unspecified whether generalized or localized, site unspecified	239	1.35%	224	10.59%
721.3--Lumbosacral spondylosis without myelopathy	304	1.71%	137	6.48%
**TOTAL Musculoskeletal Disorders**	**2899**	**16.34%**	**830**	**39.24%**
**PHYSICAL DISABILITY: Permanent injuries**				
850.0--Concussion with no loss of consciousness	547	3.08%	29	1.37%
850.5--Concussion with loss of consciousness of unspecified duration	347	1.96%	17	0.80%
**TOTAL Permanent injuries**	**894**	**5.04%**	**46**	**2.17%**
**PHYSICAL DISABILITY: Congenital anomalies**				
756.73--Gastroschisis	433	2.44%	*	*
742.4--Other specified congenital anomalies of brain	347	1.96%	*	*
756.79--Other congenital anomalies of abdominal wall	255	1.44%	0	0.00%
741.90--Spina bifida without mention of hydrocephalus, unspecified region	251	1.41%	49	2.32%
**TOTAL Congenital anomalies**	**1286**	**7.25%**	**49**	**2.32%**
**INTELLECTUAL DISABILITY**				
758.0--Down's syndrome	327	1.84%	54	2.55%
319--Unspecified intellectual disabilities	276	1.56%	152	7.19%
317--Mild intellectual disabilities	206	1.16%	97	4.59%
**TOTAL: Intellectual disability**	**809**	**4.56%**	**303**	**14.33%**
**SENSORY DISABILITY**				
3899--Unspecified hearing loss	742	4.18%	208	9.83%
365.00--Preglaucoma, unspecified	287	1.62%	36	1.70%
365.01--Open angle with borderline findings, low risk	331	1.87%	42	1.99%
389.10--Sensorineural hearing loss, unspecified	191	1.08%	35	1.65%
362.01--Background diabetic retinopathy	183	1.03%	36	1.70%
**TOTAL: Sensory disability**	**1734**	**9.77%**	**357**	**16.88%**

*values <11 are suppressed

© 2025 Dev A et al. *JAMA Network Open*.

eTable2: Most Prevalent Elixhauser Illnesses

	No disability		Only STAR+		Only ICD-9		Both STAR+ and ICD-9		Either STAR+ or ICD-9	
	n	%	n	%	n	%	n	%	n	%
**TOTAL**	**895,201**		**6,160**		**17,742**		**2,115**		**26,017**	
Congestive heart failure	792	0.09%	79	1.28%	98	0.55%	83	3.92%	260	1.00%
Valvular disease	3,913	0.44%	100	1.62%	325	1.83%	96	4.54%	521	2.00%
Pulmonary circulation disease	587	0.07%	39	0.63%	74	0.42%	39	1.84%	152	0.58%
Peripheral vascular disease	373	0.04%	42	0.68%	94	0.53%	83	3.92%	219	0.84%
Chronic pulmonary disease	46,473	5.19%	1,118	18.15%	2,512	14.16%	551	26.05%	4181	16.07%
Diabetes w/o chronic complications	28,820	3.22%	645	10.47%	1,484	8.36%	465	21.99%	2594	9.97%
Diabetes w/ chronic complications	2,109	0.24%	113	1.83%	516	2.91%	183	8.65%	812	3.12%
Hypothyroidism	15,703	1.75%	292	4.74%	768	4.33%	221	10.45%	1281	4.92%
Renal failure	744	0.08%	60	0.97%	114	0.64%	61	2.88%	235	0.90%
Liver disease	3,127	0.35%	141	2.29%	236	1.33%	131	6.19%	508	1.95%
Acquired immune deficiency syndrome	1,153	0.13%	77	1.25%	60	0.34%	26	1.23%	163	0.63%
Solid tumor w/out metastasis	793	0.09%	45	0.73%	79	0.45%	40	1.89%	164	0.63%
Coagulopthy	14,791	1.65%	141	2.29%	558	3.15%	87	4.11%	786	3.02%
Obesity	107,216	11.98%	1,289	20.93%	3,072	17.31%	579	27.38%	4940	18.99%
Weight loss	4,653	0.52%	100	1.62%	254	1.43%	54	2.55%	408	1.57%
Fluid and electrolyte disorders	43,355	4.84%	666	10.81%	2,191	12.35%	371	17.54%	3228	12.41%
Chronic blood loss anemia	166,145	18.56%	1,291	20.96%	4,075	22.97%	408	19.29%	5774	22.19%
Deficiency Anemias	127,004	14.19%	1,325	21.51%	3,558	20.05%	581	27.47%	5464	21.00%
Alcohol abuse	3,337	0.37%	173	2.81%	283	1.60%	71	3.36%	527	2.03%
Drug abuse	27,216	3.04%	850	13.80%	1,572	8.86%	294	13.90%	2716	10.44%
Psychoses	12,431	1.39%	1,490	24.19%	1,480	8.34%	547	25.86%	3517	13.52%
Depression	21,275	2.38%	896	14.55%	1,948	10.98%	481	22.74%	3325	12.78%
**Categories that overlap with disability-associated codes**										
Other neurological disorders	3,108	0.35%	181	2.94%	5,770	32.52%	672	31.77%	6623	25.46%
Rheumatoid arthritis/collagen vas	1,494	0.17%	73	1.19%	1,438	8.11%	195	9.22%	1706	6.56%
OTHER (paralysis, peptic ulcer disease, lymphoma, metastatic cancer)	116	0.01%	*	*	*	*	*	*	*	*

*values <11 are suppressed

© 2025 Dev A et al. *JAMA Network Open*.

eTable 3: Complete Regression Models for Each Outcome

Relative risk of each outcome in the mutually exclusive disability groups (95% Confidence Intervals)					
VARIABLES	**C-section**	**Severe Maternal Morbidity**	**Preterm birth**	**Low birthweight**	**Small for gestational age**
Disability Category					
No Disability	*ref*	*ref*	*ref*	*ref*	*ref*
STAR+ enrollment only	1.12 (1.08-1.16)***	2.59 (2.15-3.12)***	1.32 (1.24-1.4)***	1.36 (1.27-1.45)***	1.26 (1.15-1.38)***
ICD-9 Only	1.22 (1.19-1.25)***	4.58 (4.16-5.04)***	1.68 (1.62-1.74)***	1.77 (1.71-1.84)***	1.38 (1.31-1.46)***
Both	0.97 (0.91-1.04)	4.82 (3.96-5.86)***	1.49 (1.35-1.65)***	1.51 (1.35-1.68)***	1.43 (1.24-1.66)***
Maternal age	1.03 (1.02-1.03)***	1.05 (1.04-1.05)***	1.01 (1.01-1.01)***	1.01 (1.01-1.01)***	1.01 (1-1.01)***
Race/Ethnicity					
White	*ref*	*ref*	*ref*	*ref*	*ref*
Black	1.07 (1.06-1.08)***	1.46 (1.36-1.56)***	1.31 (1.28-1.33)***	1.65 (1.61-1.68)***	1.86 (1.81-1.91)***
Asian	0.83 (0.8-0.85)***	0.81 (0.68-0.97)**	0.94 (0.89-0.99)**	1.25 (1.19-1.32)***	1.70 (1.6-1.8)***
More than on race	1.01 (0.98-1.04)	1.04 (0.85-1.08)	1.07 (1.01-1.13)**	1.1 (1.03-1.17)**	1.10 (1.02-1.19)**
Other/unknown	0.93 (0.92-0.94)***	1.00 (0.92-1.08)	1.03 (1.01-1.05)***	1 (0.98-1.02)	0.97 (0.94-1)*
Hispanic	1.04 (1.03-1.05)***	0.81 (0.76-0.87)***	1.03 (1.02-1.05)***	1.06 (1.04-1.08)***	1.08 (1.05-1.1)***
Education					
< High school	*ref*	*ref*	*ref*	*ref*	*ref*
Completed high school	1.07 (1.06-1.08)***	1.03 (0.97-1.10)	0.98 (0.96-0.99)***	0.96 (0.95-0.98)***	0.91 (0.89-0.93)***
≥ Some college	1.11 (1.1-1.12)***	0.91 (0.85-0.97)**	0.92 (0.9-0.93)***	0.87 (0.85-0.89)***	0.79 (0.77-0.81)***
Married (Yes)	1.01 (1-1.02)**	0.96 (0.91-1.01)*	0.94 (0.93-0.96)***	0.91 (0.9-0.92)***	0.9 (0.88-0.91)***
Parity					
1	*ref*	*ref*	*ref*	*ref*	*ref*
2	1.07 (1.06-1.08)***	0.72 (0.68-0.77)***	0.96 (0.94-0.98)***	0.78 (0.76-0.79)***	0.66 (0.65-0.68)***
≥3	0.94 (0.93-0.94)***	0/78 (0.74-0.83)***	1.07 (1.05-1.08)***	0.79 (0.78-0.81)***	0.6 (0.58-0.61)***
Multiple gestation (Yes)	2.34 (2.3-2.38)***	2.38 (2.03-2.79)***	6.76 (6.64-6.89)***	8.69 (8.52-8.86)***	3.52 (3.39-3.65)***
Smoking (Yes)	1.06 (1.05-1.08)***	0.95 (0.86-1.04)	1.12 (1.09-1.15)***	1.46 (1.43-1.5)***	1.86 (1.81-1.92)***
Observations	921,218	921,218	921,218	921,218	921,218
*** p<0.01, ** p<0.05, * p<0.1					

© 2025 Dev A et al. *JAMA Network Open*.

Relative risk of each outcome in Either STAR+ or ICD-9 Group (95% Confidence Intervals)					
VARIABLES	**C-section**	**Severe Maternal Morbidity**	**Preterm birth**	**Low birthweight**	**Small for gestational age**
Disability Category					
No Disability	*ref*	*ref*	*ref*	*ref*	*ref*
Either STAR+ orICD-9	1.17 (1.15-1.2)***	4.12 (3.79-4.47)***	1.57 (1.53-1.62)***	1.64 (1.59-1.70)***	1.35 (1.29-1.42)***
Maternal age	1.03 (1.02-1.03)***	1.05 (1.04-1.05)***	1.01 (1.01-1.01)***	1.01 (1.01-1.01)***	1.01 (1-1.01)***
Race/Ethnicity					
White	*ref*	*ref*	*ref*	*ref*	*ref*
Black	1.07 (1.06-1.08)***	1.44 (1.34-1.54)***	1.30 (1.28-1.33)***	1.64 (1.61-1.68)***	1.86 (1.81-1.91)***
Asian	0.83 (0.81-0.85)***	0.81 (0.68-0.96)***	0.94 (0.89-0.99)**	1.25 (1.19-1.32)***	1.70 (1.6-1.8)***
More than on race	1.01 (0.97-1.04)	0.81 (0.68-0.96)**	1.07 (1.01-1.13)**	1.10 (1.03-1.17)**	1.10 (1.02-1.19)**
Other/unknown	0.93 (0.92-0.94)***	1.00 (0.92-1.08)	1.03 (1.01-1.05)***	1.00 (0.98-1.02)	0.97 (0.94-1)*
Hispanic	1.04 (1.03-1.05)***	0.81 (0.76-0.86)***	1.03 (1.02-1.05)***	1.06 (1.04-1.08)***	1.08 (1.05-1.1)***
Education					
< High school	*ref*	*ref*	*ref*	*ref*	*ref*
Completed high school	1.07 (1.06-1.08)***	1.04 (0.98-1.10)	0.98 (0.96-0.99)***	0.97 (0.95-0.98)***	0.91 (0.89-0.93)***
≥ Some college	1.11 (1.1-1.12)***	0.92 (0.86-0.98)**	0.92 (0.9-0.94)***	0.87 (0.86-0.89)***	0.79 (0.77-0.81)***
Married (Yes)	1.01 (1-1.02)**	0.96 (0.91-1.01)	0.94 (0.93-0.96)***	0.91 (0.9-0.93)***	0.9 (0.88-0.91)***
Parity					
1	*ref*	*ref*	*ref*	*ref*	*ref*
2	1.07 (1.06-1.08)***	0.72 (0.68-0.76)***	0.96 (0.94-0.98)***	0.78 (0.76-0.79)***	0.66 (0.65-0.68)***
≥3	0.94 (0.93-0.95)***	0.78 (0.73-0.83)***	1.07 (1.05-1.08)***	0.8 (0.78-0.81)***	0.6 (0.58-0.61)***
Multiple gestation (Yes)	2.34 (2.3-2.38)***	2.38 (2.03-2.79)***	6.77 (6.64-6.89)***	8.69 (8.53-8.86)***	3.52 (3.39-3.65)***
Smoking (Yes)	1.06 (1.05-1.08)***	0.95 (0.86-1.04)	1.12 (1.09-1.15)***	1.46 (1.43-1.5)***	1.86 (1.81-1.92)***
Observations	921,218	921,218	921,218	921,218	921,218
*** p<0.01, ** p<0.05, * p<0.1					

© 2025 Dev A et al. *JAMA Network Open*.
