# Supplementary material for: Identifying Pregnant Women With Disabilities and Maternal and Newborn Outcomes
Source: JAMA Netw Open. 2025 Mar 27;8(3):e252159. doi: 10.1001/jamanetworkopen.2025.2159 (PMC11950893; doi:10.1001/jamanetworkopen.2025.2159)
Supplement: Supplement 2. — Data Sharing Statement [file jamanetwopen-e252159-s002.pdf]

Data Sharing Statement

Dev. Identifying Pregnant People With Disabilities and Maternal and Newborn Outcomes. *JAMA Netw Open*. Published March 27, 2025. doi:10.1001/jamanetworkopen.2025.2159

Data
Data available: No

Additional Information
Explanation for why data not available: The data is protected per IRB approval and is available upon request from the Texas Department of Health and Human Services.
